# Impact of implementing the aseptic compounding management system, Medcura, on internal error rates within an oncology pharmacy aseptic unit: a mixed methods evaluation

**DOI:** 10.1136/ejhpharm-2022-003377

**Published:** 2022-10-14

**Authors:** Emily Smith, Andy Fox, Graeme Willmers, Deborah Wright, Beth Stuart

**Affiliations:** 1 Pharmacy, University Hospital Southampton NHS Foundation Trust, Southampton, UK; 2 University of Southampton Faculty of Medicine, Southampton, Southampton, UK

**Keywords:** PHARMACEUTICAL PREPARATIONS, Safety, Drug Compounding, MEDICAL ONCOLOGY, Quality Assurance, Health Care, MEDICAL ERRORS, MEDICATION SYSTEMS, HOSPITAL, PHARMACY ADMINISTRATION

## Abstract

**Background:**

As cancer survivorship improves, pressure on oncology services to provide safe, timely treatments increases. Traditional manual compounding processes are error prone, putting patients at risk. Additionally, errors have a detrimental impact on service delivery and staff morale. Information technology is increasingly utilised to improve safety and service delivery of systemic anti-cancer therapy (SACT). The compounding process control system, Medcura, was developed to manage the end-to-end process and reduce transcription and calculation errors.

**Objectives:**

To evaluate the impact of implementing Medcura on internal errors and staff perceptions of errors.

**Method:**

An aseptic process control system, Medcura, was implemented in a busy pharmacy chemotherapy production unit. Internal error and severity data were collected and analysed for 14 months before and during implementation, and 24 months after implementation. In addition, one-to-one semi-structured interviews were carried out with pharmacy staff, pre- and post-implementation. Interviews were transcribed and thematically analysed.

**Results:**

Error rates decreased after implementation from 2.9% to 2.1%. The types of error detected also changed with a decrease in worksheet and labelling errors, and an increase in assembly errors. The severity of the errors, as a percentage of total errors made, also decreased after implementation. Staff were predominantly positive about Medcura; it reduced the number of errors, eased the preparation of worksheets and labels, reduced pressure and work-related stress, and improved job satisfaction.

**Conclusions:**

Implementing Medcura has resulted in a reduction in both error rate and severity. Specifically, errors related to label and worksheet generation have seen the largest reduction. Staff have viewed these changes positively and report reduced levels of work-related stress. Further development and roll-out will improve patient safety and staff morale.

**WHAT IS ALREADY KNOWN ON THIS TOPIC**

- Internal errors occur within manual pharmacy aseptic preparation processes. They can put patients at risk and have a detrimental impact on service delivery and staff morale

**WHAT THIS STUDY ADDS**

- Implementing an aseptic compounding management system resulted in a reduction in internal errors.
- Reducing manual transcription of worksheet and labels decreased the most serious errors at this stage of the compounding process.
- Staff were positive about the change and recognised the importance of reducing errors both for the patient and their own work-related stress.

**HOW THIS STUDY MIGHT AFFECT RESEARCH, PRACTICE OR POLICY**

- Implementing an aseptic compounding management system such as Medcura can improve patient safety and staff morale.

## Introduction

### Background

Medical advances in diagnosing and treating cancer have vastly improved patient outcomes.^1^ In England and Wales, in 1971–72 24% of cancer patients survived 10 or more years post diagnosis. In 2010–11 this had more than doubled to 49.8% of patients.^2^ Additionally, development of novel systemic anti-cancer therapy (SACT—previously referred to as ‘chemotherapy’) means more people can be treated. This has resulted in increasing pressure on oncology services to provide treatments safely and on time.

In the UK, preparing SACT is predominately carried out within, or managed by, hospital pharmacy departments. It is a high risk, multi-step process, requiring aseptic compounding.^3^ Multiple checks by pharmacy staff are necessary to ensure the SACT is prepared correctly and safely.^4^

**Figure 1:**
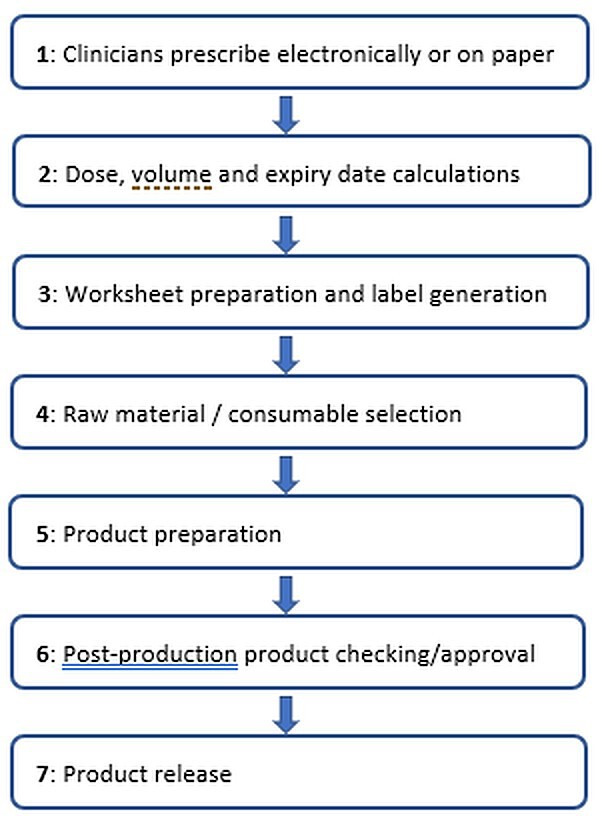
Systemic anti-cancer treatment (SACT) production workflow.

Figure 1 shows a common SACT production workflow within a UK hospital aseptic unit. The process is time consuming and error prone. Human verification is carried out to ensure the safe production and delivery of SACT.^4 5^ Irrespective of current safety measures, medication errors still occur.^5^ Analysis of the National Aseptic Error Reporting Scheme (NAERS) internal error data showed a UK error rate of 0.49%. Errors associated with SACT made up 40% of all reported internal errors, with the majority of errors associated with labelling (34.2%) and transcription (11.1%).^6^

Medication errors are defined here using the National Reporting and Learning Systems definition, and include any unintended incident relating to prescribing, preparing, dispensing, administering, monitoring, or providing advice on medicines.^7^ In the context of compounding SACT, the NAERS categories are: labelling errors, transcription errors, incorrect expiry, incorrect final volume, calculation errors, incorrect dose/strength, incorrect diluent/infusion fluid, incorrect drug, and incorrect container.^6^ Errors detected before leaving the aseptic service are classed as internal errors.

Errors can be attributed to a multitude of factors including workload, work environment and stress. Additionally, errors themselves cause more work, increased stress levels and potentially further error. Healthcare settings have previously been deemed ‘hectic, demanding, time-constrained environments’. Work environments involving frequent interruptions (eg, phone calls, pagers, or other healthcare professionals (HCPs) such as doctors, nurses and pharmacists) have been attributed to HCPs making errors.^8^ Interruptions during drug dispensing, for example, have been found to increase internal error rates by 3.42%.^9^ Similarly, a UK study which interviewed pharmacy staff about errors they had been involved in reported interruptions and distractions as frequently causing errors.^10^ This same study found that pharmacy staff more frequently attributed errors they made to a high workload. More recently a Swiss study evaluated the impact of simulated workloads on accuracy and error rates in manual aseptic preparations.^1^ Three 1 hour scenarios were designed to replicate low, medium and high workloads. Each scenario was carried out by 21 pharmacists and pharmacy technicians in a randomised order and observed by a member of the research team. In total 1007 syringes were prepared. Errors, including wrong concentration, labelling errors and selection errors, were found to increase significantly from 1.8% to 5.4% as workload increased.

In the manual processing of SACT, transcription of prescriptions onto worksheets and labels is one of the most error prone elements. For example, a root cause analysis of 401 Danish community pharmacy medication errors identified 59% of errors at the transcription stage, with the most significant of these being wrong strength or wrong drug errors.^11^

There is a drive in the UK to evaluate and implement technology to improve workflow safety in the preparation of SACT.^12^ Research exists where technological advancements in compounding workflow systems and robotics have been made. However, these areas of research have focused on improvements to the final product compounding time.^13–16^ They do not evaluate the impact on errors from moving from paper to computerised processes, akin to the implementation of electronic prescribing. In addition, there are few studies which have looked at the views of staff relating to the adoption of new technologies, and none which use qualitative interviewing methodology to explore views in depth.

### Impact of errors

Undetected errors put patients at risk of serious harm.^11^ Additionally, errors can directly or indirectly have an impact on staff.^17 18^ Involvement in errors can result in increased stress levels, decreased job satisfaction,^19 20^ and subsequent increases in sick leave and staff turnover. Workplace stress is ‘a major cause of occupational ill health, poor productivity and human error’.^20^ The Health and Safety Executive reported an estimated 1.4 million work-related ill-health cases in 2018/19. Of these, 44% were related to stress, depression or anxiety resulting in 12.8 million lost working days. In addition, recording, reporting and rectifying errors takes time, affecting workflow and slowing down service delivery, with community pharmacists reporting that patients become frustrated when there is a delay.^21 22^

The cause and impact of errors can become cyclical due to a switch of focus on error correction rather than prevention. Two UK based qualitative studies explored community pharmacists’ experiences of errors, work environment, workload and stress levels.^23 24^ A number of the pharmacists interviewed considered the implications of these factors on increased risk of error: ‘so much pressure on the staff they stress them out, pressure and stress cause errors’.^23^

### Development of a solution

It is clear that the use of IT has been shown to improve safety—specifically, the introduction of computerised physician order entry systems has reduced medication errors.^25–27^ The aim of this study is to evaluate the impact of implementing the bespoke aseptic comounding management system (ACMS), Medcura, on internal errors and staff perceptions of errors.

## Methods

### Setting

The evaluation took place in an 1100 bed teaching hospital in the south-east of England. The hospital is a regional centre for cancer care. The oncology pharmacy department consists of 28 staff, within a larger pharmacy department, who deliver clinical and production services to approximately 2000 patients per month preparing approximately 2500 SACT doses per month.

### Description of system

With the aim of improving SACT production and delivery to patients, pharmacists at University Hospital Southampton in conjunction with software design specialists developed and implemented Medcura, a ‘process control system’ designed to manage the end-to-end compounding process and delivery of SACT. The modular system incorporated: pharmacist order entry, dose and expiry calculations, pre-populated worksheet and label production, picking lists, and the ability to schedule work. These processes were identified as carrying the highest risk of error and correspond to steps 2–4 in figure 1. The system did not include any in-process checks such as gravimetric controls or video/remote checking, although it has the capacity to interface with such systems in the future. Currently in-process checks are documented on the pre-populated paper worksheets produced by Medcura. At the time of writing, pharmacists transcribe SACT orders from the electronic prescription into Medcura until system integration is developed. Medcura does, however, link to the electronic patient record ensuring those patient details are accurate and providing access to previous treatment regimens. Medcura has the ability to integrate with robotic and gravimetric software once these aspects have been developed further.

### Implementation

Medcura was implemented in phases beginning in October 2016 with three small inpatient wards, followed by three further wards in January 2017. In February 2017, in the largest patient group, outpatients’ orders began to be processed using Medcura. Finally, paediatric chemotherapy was added to the system in April 2017. During each of these phases of implementation, a proportion of orders were processed using the original method and Medcura simultaneously, with both types of worksheets being used in parallel to ensure the accuracy of Medcura. Continuous data monitoring and tracking of internal, department generated, data was undertaken.

### Study design

This pre- and post- implementation service evaluation followed a convergent parallel design.^28^ Here both quantitative and qualitative data collected concurrently are used to provide a greater understanding of the impact of implementing Medcura on internal errors in oncology pharmacy associated with compounding SACT. This work was categorised as a service evaluation by the research and development department, therefore ethical approval was not sought.

### Quantitative data collection and analysis

Internal error data are routinely and spontaneously recorded during the preparation and production process and categorised following the NAERS definitions (table 1).^6^ At the time of the study any error identified by staff at any stage of the process was recorded on a paper-based collection tool. The data were then entered into standard spreadsheet software for further analysis and trending. The level 1 definitions are shown in table 1. Each of these definitions is further subdivided into highly specific level 2 errors such as wrong expiry on the worksheet. This study evaluated 8 months of pre-implementation error data (February 2016 to September 2016), 6 months of implementation data (October 2016 to April 2017), and 25 months of post-implementation data (May 2017 to May 2019). The proportion of each error type and error severity score was calculated for each month from February 2016 to May 2019. Means were then calculated for each of the three implementation phases (pre, during and post). An error severity score was determined for each error type by a process of consensus scoring. Seven experienced members of pharmacy staff were asked to independently score each level 2 error type between 0 (least severe) and 5 (most severe), depending on how severe the consequence of the error would be should a product made with an error reach a patient. A median value of the scores was used as the severity score for each error.

**Table 1:** NAERS error types and grouping for analysis

Error type as detailed by NAERS^6^	Error types grouped for analysis
A. Prescription errors	A. Prescription errors
B. Worksheet preparation errors	B. Worksheet preparation errors
C. Label generation errors	C. Label generation and packaging errors
D. Labelling and packaging errors	D. Assembly and ancillary item errors
E. Assembly errors	E. Product preparation errors
F. Production preparation errors	F. Product approval errors
G. Ancillary item errors	
H. Product approval checking errors	

NAERS, National Aseptic Error Reporting Scheme .

For the purpose of this analysis, due to the low numbers of errors, ancillary item errors and assembly errors were combined. The labelling and packing errors were also combined with the label generation errors.

### Qualitative data collection and analysis

A pre- and post- implementation qualitative evaluation was designed to provide a more in-depth understanding of staff views relating to oncology pharmacy processes used pre- and post- implementation. The methodology has been credited with providing a richness of data not possible through the use of questionnaires.^29^

One-to-one semi-structured interviews with oncology pharmacists, pharmacy technicians, and pharmacy support workers (PSWs) were conducted at two time points by the main investigator (ES). Interviews were designed to elicit what knowledge, views and experiences were held relating to the work procedures, processes and environment, in the oncology pharmacy before and after the implementation of Medcura. The first phase of interviews (pre-implementation) took place between June and September 2015 (n=25), and the second phase (post-implementation) took place between June and October 2017 (n=19). All staff were invited to participate in both phases. Interviews in both phases lasted between 30 and 90 min. Interviews were audio recorded, transcribed verbatim, anonymised and entered into Nvivo 10 to allow for thematic analysis to be carried out.^30^ The analysis presented here focuses on staff views and experiences relating to internal errors and the impact Medcura had on the internal error rates, error types, and error severity.

## Results

Over the evaluation period, the total number of SACT items made was 101 726 with a total of 2461 internal errors.

### Error rates and error severity

The mean number of errors per month, overall error rates, and workload for the three evaluation periods were calculated and are shown in table 2. The mean monthly workload was similar across the three evaluation periods. The mean error rate remained stable at 2.9% and 3.0% during pre-implementation and implementation phases. The mean error rate then dropped to 2.1% post-implementation. Table 2 also shows the breakdown by both error type and processing system.

**Table 2:** Workload and error rates pre-, post- and during implementation

	Pre-implementation (8 months)	Implementation (6 months)	Post-implementation (25 months)
	Mean (SD)	Mean (SD)	Mean (SD)
Monthly errors	77.9 (41.7)	75.3 (9.0)	52.4 (17.1)
Monthly error rate	2.9%	3.0%	2.1%
Monthly workload	2652.3 (118.5)	2488.3 (147.6)	2523.6 (290.0)

Error type	Mean* (SD) %†			Mean* (SD) %†			Mean* (SD) %†		
Prescription	0.3	(0.5)	0.4%	0.3	(0.5)	0.4%	1.0	(1.8)	1.6%
Medcura	–			0.1			1.0		
Paper	0.3			0.1			0.1		
Worksheet preparation	8.3	(8.3)	9.0%	8.3	(3.6)	11.0%	4.2	(2.4)	8.1%
Medcura	–			0.4			0.4		
Paper	8.3			7.9			3.9		
Label generation and packaging	51.3	(26.7)	65.7%	41.3	(17.9)	53.6%	14.6	(7.8)	27.8%
Medcura	–			0.4			1.0		
Paper	51.3			40.9			13.6		
Assembly and ancillary items	14.1	(8.9)	18.4%	20.0	(10.6)	27.6%	28.5	(12.3)	54.4%
Medcura	–			6.3			23.3		
Paper	14.1			13.7			5.2		
Product preparation	3.6	(2.8)	6.2%	5.1	(3.4)	7.1%	2.8	(2.7)	5.9%
Medcura	–			1.6			2.3		
Paper	3.6			3.6			0.6		
Product approval	0.3	(0.7)	0.2%	0.1	(0.4)	0.2%	0.5	(0.9)	0.9%
Medcura	–			0			0.3		
Paper	0.3			0.1			0.2		

*Mean of monthly errors; values rounded to 1 decimal point.

†Proportion of combined monthly errors (%).

All error types except prescription and assembly errors showed a decrease in the mean error rate per month. Labelling errors showed the largest decrease from 51.3% per month to 14.6%. Assembly errors increased from 14.1% per month to 28.5%. Error rates using the paper-based systems remained higher throughout the evaluation with the exception of assembly errors.

Data relating to the severity of errors are shown in table 3. Following the severity scoring process there were no error types with a severity score of 5. There was a decrease in the most severe errors (those with a severity score of 2, 3 and 4) while the errors scoring 0 and 1 increased slightly. There was also an emergence of errors requiring a categorisation of ‘other’ due to the introduction of errors not listed by the NAERS at level 2. Many of these were errors related to data that required manual entry on to the worksheet, such as batch numbers of consumables. It was therefore not possible to assign these with a severity score for this analysis.

**Table 3:** Error severity scores pre-, post- and during implementation

	Pre-implementation (8 months)			Implementation (6 months)			Post-implementation (25 months)		
	Median (LQ, UQ)			Median (LQ, UQ)			Median (LQ, UQ)		
	2 (1, 2)			2 (1, 2)			2 (1, 2)		
	Mean* (SD) (%)†			Mean* (SD) (%)†			Mean* (SD) (%)†		
Severity score 0	4.0	(5.4)	4.7%	7.7	(6.6)	10.9%	9.7	(3.6)	18.8%
Medcura	-			3.3			7.3		
Paper	4.0			4.4			2.4		
Severity score 1	27.0	(16.7)	34.0%	23.6	(9.1)	30.6%	12.5	(6.0)	23.9%
Medcura	-			1.9			6.1		
Paper	27.0			21.7			6.4		
Severity score 2	37.0	(17.5)	48.4%	29.9	(7.8)	39.5%	17.6	(6.7)	33.5%
Medcura	-			1.3			7.5		
Paper	37.0			28.3			10.1		
Severity score 3	7.8	(6.3)	8.9	7.3	(3.2)	9.6	4.6	(3.4)	8.4
Medcura	-			0.4			2.7		
Paper	7.8			6.9			2.0		
Severity score 4	2.0	(1.5)	3.8%	1.6	(1.8)	2.0%	0.6	(0.9)	1.0%
Medcura	-			0.1			0.4		
Paper	2.0			1.4			0.2		
Other	0	(0)	0	5.1	(2.7)	7.2%	6.7	(3.7)	12.9%
Medcura	-			1.6			4.3		
Paper	0			3.6			2.4		

*Mean of monthly errors; values rounded to 1 decimal point.

†Proportion of combined monthly errors (%).

LQ, lower quartile; UQ, upper quartile.

### Staff perceptions of internal errors and error severity

Interviewees consisted of pharmacists (11), pharmacy technicians (13) and pharmacy support workers (11). Nine of the participants took part in both pre- and post- implementation interviews

Before the implementation of Medcura the general view among staff was that, while there was the chance of error at every stage, the majority would occur before the compounding phase and were most likely to be worksheet or label generation errors. These included: selecting the wrong worksheet or label from the template files, making an error when calculating the drug expiry time and/or date, making a spelling error in patient details when hand transcribing from prescription to worksheet and/or label, product batch number being incorrect, product batch numbers not matching labels, and other general transcription errors. Labelling was particularly noted to be a frequent source of error:

“So that is one of our main things […], labelling, transcription of prescriptions and you know, it actually going through the clean room and coming out the other side with a label error on it.” (ID1501)

These were frequently attributed to the busy and noisy work environment in combination with the manual process required to complete the tasks:

“I seem to make the most errors in document prep, […] not because I can’t do the role but just because I find it more stressful and there’s too much going on for me to be able to keep up with everything so I probably… that’s when I think I miss things…” (ID1509)

The assembling of products for the compounding process was deemed to work well and was viewed to be the most organised step in the compounding process:

“That’s actually one of the most organised, I’d say. Like the sheets come in and you just, yeah. You just get everything out, get everything ready, you’ve got the checkers there. I’d say that’s fine. That’s probably the most organised area.” (ID1513)

Despite being viewed as a well organised part of the process, product assembly before compounding was also reported to be an area where a large number of errors would occur. These were associated with setting up the wrong ancillary components (eg, the wrong type or number of syringes or wrong diluent bag).

Many of the staff talked about the cause of the majority of internal errors being down to ‘just human error’:

“We’re all human, we all make mistakes no matter what grade you are.” (ID1513)

### Post-implementation

Overall there was agreement among all staff groups that the implementation of Medcura had resulted in a noticeable reduction in internal errors within oncology pharmacy. Most noted was a reduction in errors relating to the generation of worksheets and labels:

“So old worksheets will be, like, wrong patient details, wrong patient batch numbers—so, sometimes the batch number will be wrong—the expiry times can be wrong, the actual drug concentrations are wrong because people can put the numbers the wrong way around, or the wrong order, so there’s significantly more errors on the old worksheets than the new ones.” (ID17330)

This change was viewed by staff to not only reduce errors, but reduce staff work-related stress and improve job satisfaction:

“For document prep people I think it would be more satisfaction for them using Medcura because they always felt like all the errors are falling on them because of the transcription and distractions and everything else out there.” (ID1507)

With the implementation of Medcura also came a change to the working procedures, with the chemotherapy orders now being put on the system by the pharmacists at the point of prescription screening, rather than being handwritten by technicians and PSWs. This was viewed by pharmacy staff as beneficial in that Medcura ‘does the calculations for you’ (ID1727). Technicians and PSW’s described having to ‘trust it gets put on right in the first place’ (ID1509):

“Trusting that, you know, the computer has done everything that it should’ve done correctly, obviously, there is much less scope for decimal point being in the wrong place or somebody miscalculating something.” (ID1508)

Technicians felt that if the product was put onto the system incorrectly at the beginning, the error could go unnoticed until the end of the production process which could cause delays to the patients’ treatment:

“It gets put on (Medcura) by the pharmacist and then it doesn’t—the prescription doesn’t get reviewed ‘til the, kinda, end stage, there could be that risk that something is made incorrectly and not picked up until the final point, so there could be a delay to treatment for patients.” (ID1731)

Product assembly was an area where staff still felt errors occurred:

“Our errors have shifted somewhat from, sort of, more labelling data entry, with the implementation of Medcura, onto the assembling.” (ID1524)

These errors mostly related to assembling the wrong type or number of syringes or wrong diluent:

“It can be quite easy to pick, like a syringe instead of a bag, or the wrong-sized bag, or to not pick the right bag.” (ID1507)

It was felt that these errors were down to human error rather than Medcura:

“The error side is more when they’re in the room setting it up, I would say. I wouldn’t say it’s to do with the Medcura system. It’s more human error than the system error.” (ID1730)

However, one suggested reason for the occurrence of errors at this stage was that not all items needed for making the products were listed in the Medcura worksheet ‘picking lists’; sometimes they would just be noted in the compounding instructions which were on a separate page. But ultimately these errors came down to the whole worksheet not being read during assembly because staff ‘think they know’ (ID1513) the method and necessary items to be assembled.

Finally, the ‘making’ and ‘releasing’ stages were perceived to have a lower frequency of errors than some of the other stages:

“When you’re making, you’re just doing what it tells you, so there must be a lot less errors.” (ID1505)

“There’s not much pressure on releasing, [be]cause most of the stuff on Medcura, as long as it’s not been uploaded wrong, there’s never really any errors coming through that way.” (ID1732)

### Error severity

A small number of staff also discussed their views on the severity of the errors that occurred. It was felt that although errors still occurred, generally these were less serious errors than before the implementation of Medcura.

“My overall perception is I think the percentage of errors in general probably hasn’t changed, but I think that the critical to more operational has, so, from a patient safety perspective, I think that the number of errors that would affect the patient has been reduced.” (ID1524)

### General feelings

Overall, staff displayed a degree of trust in the Medcura system because of the validation it had undergone. Subsequently, this means that safety was improved because fewer mistakes/errors were being made.

## Discussion

Implementing the ACMS, Medcura, has reduced overall internal error rates within an oncology pharmacy aseptic unit attached to a regional centre for cancer care. While the reduction is not statistically significant, it does represent an evident operational change. The largest reduction was seen in errors related to the generation of worksheets and labels. Before implementation, completion of these processes was performed manually by an operator using MS Word templates. The removal of the transcription process resulted in almost the complete removal of worksheet and labelling errors. Conversely, there was an increase in assembly errors. This was due to the reduction in flexibility over consumable choice brought about by the introduction of the ACMS when selecting, for example, the syringe size to use when a range would previously have been acceptable. The system forced a specific syringe type which took the staff time to get used to as part of their work. Mis-selection of the designated syringe size was regarded as a low severity error. Additionally, there was an increase in errors recorded as ‘other’ which did not fall into any of the NAERS error categories and varied too much to allow for a severity score to be attributed during the consensus scoring.

The potential harm of each error was assessed using an internally derived severity scale. Following the implementation of Medcura, there was a reduction in the occurrence of errors with severity scores of 1 to 4 (4 being the most sever error). These are errors which if they reached the patient would have the greatest level of harm. Only those errors with a severity score of zero increased. These low severity errors are primarily related to the assembly process and include selecting the wrong size or number of syringes to prepare a product. These present no risk to the patient and are easily identified and resolved. The errors coded as ‘other’ during severity consensus scoring were reviewed by quality assurance and the research team and also deemed to be non-severe and no risk to the patient.

Thematic analysis of staff interviews showed that pre-implementation, staff felt errors could occur at any time. They felt manual processes involved in preparing for and compounding intravenous (IV) SACT within a disruptive work environment, particularly transcribing worksheets and labels, were the main contributors to internal errors. Staff generally agreed that the implementation of Medcura had prevented the number of errors they saw and were involved in. The exception to this was the post-implementation increase in assembly errors. However, they felt that the errors that did still happen were less severe than those before implementation. The biggest changes for staff were related to preparing worksheets and labels — the process became easier with the possibility of making a transcription error removed from items processed using Medcura. Staff also felt that their work-related stress within the department had reduced and job satisfaction had improved. There was no longer the pressure associated with having to redo a label or remake a product. They trusted the system which now did all the calculations of volumes and expiry dates for them.

Healthcare professionals and organisations are increasingly looking to technology to improve safety through minimising the risk of human error.^4^ Certainly electronic prescribing systems have shown significant reductions in prescribing errors.^31^ However, the system has introduced errors that did not previously exist much, as Wright *et al* 4 found when they implemented an IV workflow management system and compared it to a baseline manual process. They were able to detect more errors overall in addition to detecting new types of errors, meaning the process was safer with fewer errors reaching patients, much like implementing Medcura.

A major benefit of the implementation of Medcura has been the streamlining of the process making workflow smoother, and the decrease in errors means less time spent re-working a product made with an error (ie, wrong dose, wrong label, etc). Correcting errors takes time, adding to the workload burden and staff stress.^9^ To the authors’ knowledge this is the first report containing an analysis of the views of pharmacy staff to such a change in their practice. These views indicate clearly the impact of a perceived positive change in the working processes.

### Why do errors still occur?

Medcura has not yet been able to remove all errors, for two main reasons. First, while the majority of the work is processed through Medcura, a small number of complex products (eg, doxorubicin-eluting beads) are still processed manually. Clinical trials are also not yet fully implemented on Medcura. However, as of June 2020 the clinical trials module was complete and new clinical trials were initiated using the Medcura process, thus reducing the risks of manual processing error further. Second, there remains some form of human interaction with the process, whether it is entering the prescription onto the system or assembling the required components needed for compounding each item. Human error ‘involves unintentional and unpredictable behaviour that causes or could have caused an undesirable outcome’.^32^ In terms of prescription entry, we intend to explore a link to our electronic prescribing system in the future, to include an automatic stop before passing through to allow for a clinical screening step by the pharmacist.

## Limitations

The data from which error rates were calculated were collected by many different pharmacy staff throughout the course of the study period. This followed the NAERS reporting system whereby errors seen are recorded, collated and reported locally and nationally to help understand and improve the services. However, a variance in the reporting of errors was likely. Some errors viewed by or involving staff were not reported in this way. This could have been due to a lack of time to record them, meaning to record the error but then becoming distracted and forgetting, or perceiving the error to be of low severity and therefore not necessary to report. Both would result in the data not fully reflecting all the errors that took place. Further comparison of errors as clinical trials move over to the Medcura system will demonstrate further the benefits of the ACMS. The study was not powered to detect differences over time in the error rates, and formal hypothesis testing about the effects of the implementation on error rates has not been undertaken. At present there are insufficient data points for a formal time series analysis, but we hope that with ongoing data collection this is something that can be explored further in the future. Finally, it would have been beneficial to have interviewed more staff at both pre- and post-implementation; unfortunately, due to the time taken to implement the system alongside staff leaving or joining between time points, this was not possible.

## Conclusions

Implementing Medcura has resulted in a reduction in the overall error rate with a notable reduction in worksheet and label generation errors. Post-implementation errors that continue to occur were rated as less severe than pre-implementation errors. The only error types that increased were assembly errors of low severity. The reduction in overall errors was viewed positively by staff, with reported work-related stress decreasing due to improved workflow through not having to redo worksheets, labels or products as a result of errors, saving them real time. These views clearly show the importance of continuing to develop the system and further reduce errors.

## Data Availability

Data are available upon reasonable request.
